# A Practical Treatise on Lepra Vulgaris; to Which Are Added, Observations on the Treatment of Some of the Local Varieties of Psoriasis

**Published:** 1835-01-01

**Authors:** 


*A Practical Treatise on Lepra Vulgaris; to which are added, Observations on the Treatment of some of the Local Varieties of Psoriasis.* By Edward Beck, m.d.—Ipswich. 8vo. pp. 74.

The common remedies prescribed for the cure of lepra are, as our readers well know, dulcamara and arsenic. Our author has tried them both, but without being satisfied with either; and he objects to the latter and more potent medicine, first, that it is dangerous in the doses which are required for the cure of the disease; and, secondly, that this cure is not permanent. He has therefore adopted a different set of remedies, and, as he informs us, with great success.

If the disease is of long standing, and there is considerable pain and inflammation, Dr. Beck, before commencing what he calls his "specific plan," premises a few doses of aperient medicines; such as equal parts of Pil. Rhei c., and Extr. Colocynth. c., with or without the addition of blue pill. In such cases, too, half a drachm of precipitated sulphur, with five grains of the dried subcarbonate of soda, two or three times a day, is found to reduce the irritation. The best local applications are the Liq. Plumbi Subacet. dil., or "a lotion containing the Plumb. Subacet. in combination with Zinci Sulphas." (P. 22.) This latter lotion can hardly be said to contain a combination of the two metallic salts, for they decompose each other, forming a solution of acetate of zinc, in the manner directed in the Edinburgh Pharmacopœia; but the lotion should be filtered, to separate the insoluble sulphate of lead. When the irritation has been mitigated by these means, the specific remedies may be prescribed; and, in mild cases, the treatment may commence with them. They consist of tar, exhibited externally and internally, in the following manner.

"The specific plan consists in applying the following liniment to the parts affected:—R. Picis Liquid. Sulphuris. Adipis Præpar. sing. unciam. misce.

"The following pills are at the same time to be taken regularly:

—℞. Picis Liquidæ unciam dimidiam. Flor Tritici q. s. misce. et divide in Pilul. sing. gr. v. iii. ad vi. ter in die sumantur.

"Where the irritation is very considerable, a milder liniment may first be had recourse to, as the following:—℞. Picis Liquidæ. Sulphuris singul. unciam dimidiam. Adipis Præparat. unciam. misce." (P. 24.)

As these ointments are stimulant, it is requisite, in cases where the irritation is considerable, not only to begin with the milder one, but to let it remain on the skin for a minute or two only. It is likewise requisite to continue the use of the pills for some time after the disappearance of the eruption. To prevent the pills being tasted, (as they are then apt to cause nausea,) the best method is to take them "at the point of a tablespoonful of gruel."

Dr. Beck tells us that "two months will be required for the cure of lepra in its advanced stage; and, where it has been of very long standing, it may even be prudent to continue the pills somewhat longer." (P. 32.)

We will now give an abstract of our author's cases.

Case i. A patient, who had been treated for several months in the manner recommended by Willan and Bateman, but who, far from improving, was getting worse, came under our author's care, suffering from lepra in a very violent form. After trying a variety of remedies for ten days, the Liquor Arsenicalis and Decoct. Dulcamaræ were prescribed, and persevered in for nearly three months, with great advantage. They were now omitted; and, after exposure to cold and wet, the disease returned in an aggravated form. The medicines were resumed, but in vain. A strong solution of the nitrate of silver was now applied, to a small part of the arm, with much benefit, and the tar ointment and pills being soon after exhibited, a complete cure was effected in a few weeks. This patient has since had some slight returns of the eruption, which have been immediately removed by the use of the pills.

Case ii. S. W., æt. sixty-one, who had tried a variety of treatment without benefit, applied for advice, in consequence of one leg being enveloped in a thick crust, from just below the patella to the instep. The disease was so far advanced, that its characteristic marks were no longer obvious, and our author did not recognise it till his third visit, when a new leprous spot was visible. This patient soon got well under the use of the specific.

Case iii. H. L., æt. eleven, had suffered from lepra for six years. In this case, Dr. Beck says, "I prescribed for this patient fifteen grains of Sulph. Præcip., with five grains of Soda Exsic. twice in the day, and a dose of the pills, with Extr. Coloc. comp., and Pil. Rh. comp., three grains of each, every second night." (P. 45.) We do not know what is meant in this passage by "the pills:" we should have understood the tar-pills, were it not that our author says, in the very next sentence, "as there was little local irritation in this case, the stronger Ung. Picis comp., after a few days, was applied to the arms, legs, and head, but no Pil. Picis were prescribed."

We must observe, however, that this little work is somewhat carelessly written: in several places Dr. Beck calls his ointment a liniment, and in others gives this name to a cerate of camphor. Then, too, at pp. 22 and 23, he has allowed his printers to put *granas* for *grana:* but, instead of dwelling on these niceties, we will return to our analysis of the case.

The tar-ointment alone was first used: this cured the arms, and benefited the legs, but scarcely affected the leprous eruption on the head. Dr. Beck then ordered the head to be dressed alternately with the Ung. Picis comp., and the Ung. Hydrarg. Nitr. This improved the state of the head; but, as the disease returned on the fore-arms, our author thought it necessary to administer the tar-pills, which completed the cure. A slight eruption of a suspicious character subsequently appeared on the fore-arms: for some weeks nothing was prescribed, and there was no alteration in the disease; but it was then removed by the Ung. Hydr. Nitr.

CASE IV. The patient, a young woman of seventeen, was suffering from the disease, in its acute form. After purging for a week, the milder ointment and the pills were prescribed with great advantage; yet it was necessary, in order to effect a complete cure, to use an ointment more tarry even than the strong one given above; this being the only case in which our author has ever found so stimulating an unguent requisite. The following is the composition of the Ung. fortiss.

℞. Sulph.

Adip. Præpar., aa ℥ss.

Picis liquidæ, ℥j.

CASE V. This patient had suffered from slight attacks of lepra for several years, but it had not affected the scalp till within a few months. He did not wear clothes enough to keep him warm; an absurdity which Dr. Beck has remarked in most of the leprous patients who have come under his care. We will give the treatment in our author's own words.

"The treatment in this case varied but little from that pursued on other occasions. The pills, composed of equal parts of Pil. Rhei comp., and Extr. Coloc. comp., were given a few days, and the Liquor Potassæ, in doses of twenty drops, three times in the day.

"The stronger Ung. Picis comp. was soon applied to the few patches on the lower extremities, and the Pil. Picis Liquidæ were directed to be taken, and increased to eighteen daily.

"To the head, the Ung. Hydrarg. Nitrat. was applied alternately with the Ung. Picis. comp.; the hair being kept as closely cut with scissors as possible.

"In this, as in other cases under my care, the disease on the scalp proved more obstinate than on the limbs, and continued to require attention some little time after it appeared perfectly well elsewhere.

"The following ointment was, after a time, substituted for the alternate use of the Ung. Hydrarg. Nitrat., and Ung. Picis comp., and with most satisfactory results:—℞. Picis Liquidæ, Sulphuris Ung. Hydrarg. Nitrat. sing. unciam. dimid. Misce.

"The use of flannel and warm clothing generally was manifestly beneficial to the general health, and aided powerfully, I am persuaded, the other means for removing the disease." (P. 53.)

Case vi. The disease in this instance was of a very acute form, and seems to have been produced by eating plentifully of codfish, for several successive days.

"In this case, the following was the plan of treatment adopted. An active aperient of Pil. Rhei. comp., and Extr. Colocynth, comp. was given every second night, and the Sulphur. Præcipit. in combination with the Sodæ Subcarb. Exsicc., was taken three times in the day.

"After a few days, the heat and irritation being much moderated, the powders and pills were discontinued, and the Infusum Rosæ, containing a drachm of Magnesiæ Sulphas in each dose, was substituted. In ten or twelve days the disease was on the decline; and, in three weeks from the attack, it was perfectly removed.

"I have met with two other instances of lepra being occasioned by errors of diet, not very unlike the foregoing. In one case, the individual had partaken plentifully of cucumber; and in the other case, of walnuts.

"The Pilul. Picis Liquid., and the Unguent. Picis. comp., would, I am persuaded, greatly aggravate all the symptoms in cases of this kind. This form of lepra, unlike the other, has an evident tendency to spontaneous cure, and will disappear in two or three weeks, under common antiphlogistic treatment, attention being paid to the state of the stomach and bowels." (P. 55.)

Our author remarks, in his recapitulation, that asthma sometimes appears to alternate with lepra; and that, in such instances, it would probably be injurious to repel the latter by astringent applications, unaided by internal remedies.

Dr. Beck's essay on *Psoriasis* is short, but bears marks of careful observation; yet we hardly understand the distinction he draws, when he says that "the parts occupied by the eruption of psoriasis have a rough chapped appearance, and somewhat resemble lepra, if looked at superficially only; but, when attentively examined, and with the aid of a magnifier, the difference is very striking. This rough appearance is not occasioned by scales, as in lepra, but by numerous partially detached, minute portions of cuticle." (P. 63.) Surely "partially detached minute portions of cuticle" are scales. Bateman, too, puts psoriasis, as well as lepra, among the scaly diseases.

After the preliminary use of aperients and tepid ablution, our author employs a cerate, composed of half an ounce of camphor to two ounces of Cerat. Cetacei, and the following powder:

℞. Sulph. Præcip., gr. xxad xxx.

Sodæ Subcarb., gr. vj.

Ft. pulv. nocte et manè sumendus.

The quantity is to be such as to relax the bowels; but, if the patient dislikes the powder, the Liquor Potassæ may be substituted, and the bowels kept open by the Pil. Rhei c., with or without a grain or two of blue pill; but the powder is more efficient. After a few days, the strength of the camphorated cerate may be increased. Three cases are given at length, in which these remedies were employed.

Case i. A lady, æt. sixty, had suffered from psoriasis palmaria for several years, and, during great part of the time, from severe dyspepsia. The disease was much mitigated in a short time by the plan of treatment just mentioned; the Pulv. Rhei c. and the Liq. Potassæ being substituted, after some time, for the powder.

Case ii. D. S., æt. thirty, had suffered from psoriasis labialis, and dyspepsia, for several months. The Decoct. Aloës c., with magnesia, the powder, and the camphorated ointment, ultimately effected a cure, though the case was the most obstinate that Dr. Beck has ever seen.

Case iii. G. L., a gardener, aged about forty, had suffered from psoriasis diffusa for several years. "Nausea, heat, and thirst, with a general feeling of indisposition, were complained of, as well as want of appetite and restlessness at night. The tongue was much loaded, and the breath very offensive, as I have generally observed in psoriasis. The treatment in this case was antiphlogistic for several days, with frequent ablution of the parts, which were much inflamed and painful, with warm decoction of bran."

(P. 73.) The feverishness having been moderated in this manner, a perfect cure was soon effected by the powder and cerate.

This occurred three years ago: a relapse has since taken place, but the disease speedily yielded to the same treatment.

Dr. Beck's treatment of lepra is, we believe, quite new as regards the pills; a tar-ointment has already been recommended. "As external applications," says Mason Good, "most benefit appears to be derived from the tar-ointment, as employed by Dr. Willis, and a dilute solution of sublimate, or the unguentum hydrargyri nitrati, as recommended by Dr. Willan." (*Study of Medicine*, vol. iv. p. 455, fourth edit.) It is also among the remedies advised by Bateman. (*Practical Synopsis*, p. 33, sixth edit.)

In the treatment of psoriasis, too, Bateman says, "if the constitutional disturbance has subsided, the use of the fixed alkali, combined with sulphur lotum, or with an infusion of cinchona, together with tepid washing with simple water, or milk and water, will gradually remove the complaint." (*Pract. Synopsis*, p. 44, sixth edit.)

But, even if the tar-pills likewise had been mentioned among the remedies against lepra, we should not deny Dr. Beck the merit of having selected a new, as well as a successful medicine, in the treatment of an obstinate disease; for, in a tentative art like ours, where almost every remedy has been proposed, if not tried, against every malady, it would be hard to refuse the praise of novelty to him who acts where others talk, and succeeds where his predecessors have despaired.

It now merely remains to be seen whether this medicine will be equally powerful in the hands of other practitioners. We trust that it will be so; and that it may be our office to congratulate our readers on having obtained another trustworthy therapeutic agent, and Dr. Beck, on the enrolment of his name among those who have advanced the practice of physic.

